# Crystal structure of 3-[4-(pyrimidin-2-yl)piperazin-1-ium-1-yl]butano­ate

**DOI:** 10.1107/S1600536814018972

**Published:** 2014-08-30

**Authors:** Thammarse S. Yamuna, Jerry P. Jasinski, Manpreet Kaur, Brian J. Anderson, H.S. Yathirajan

**Affiliations:** a Department of Studies in Chemistry, University of Mysore, Manasagangotri, Mysore 570 006, India; b Department of Chemistry, Keene State College, 229 Main Street, Keene, NH 03435-2001, USA

**Keywords:** crystal structure, 3-(piperazin-1-ium-1-yl)butano­ate, zwitterionic form, fused heterocyclic derivatives, aza-Michael reactions

## Abstract

The title compound, C_12_H_18_N_4_O_2_, crystallizes in the zwitterionic form with protonation at the N atom of the piperazine ring bearing the carboxylate group. The piperazine ring adopts a slightly distorted chair conformation. In the crystal, N—H⋯O hydrogen bonds are observed, forming chains along [010]. The packing is consolidated by C—H⋯O inter­actions, which generate a three-dimensional network.

## Related literature   

For general background and pharmacological properties of fused heterocyclic derivatives, see: Amin *et al.* (2009 ▶); Ibrahim & El-Metwally (2010 ▶); Kuyper *et al.* (1996 ▶); Onal & Yıldırım (2007 ▶); Padmaja *et al.* (2009 ▶); Tollefson *et al.* (1991 ▶). For pharmacological properties of pyrimidines, see: Burdge (2000 ▶). For background to aza-Michael reactions, see: Arend *et al.*(1998 ▶); Vicario *et al.* (2005 ▶); Xu & Xia (2005 ▶). For related structures, see: Jin *et al.* (2012 ▶); Parvez *et al.* (2004 ▶); Yamuna *et al.* (2014*a* ▶,*b* ▶).
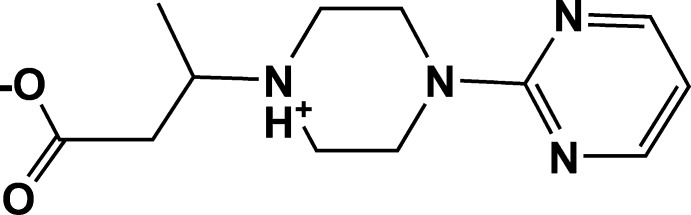


## Experimental   

### Crystal data  

C_12_H_18_N_4_O_2_
*M* *_r_* = 250.30
Monoclinic, 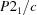

*a* = 13.5157 (6) Å
*b* = 7.8454 (3) Å
*c* = 12.2147 (5) Å
β = 106.884 (5)°
*V* = 1239.36 (9) Å^3^
*Z* = 4
Cu *K*α radiation
μ = 0.77 mm^−1^
*T* = 173 K
0.32 × 0.22 × 0.06 mm

### Data collection  

Agilent Eos Gemini diffractometer
Absorption correction: multi-scan (*CrysAlis PRO* and *CrysAlis RED*; Agilent, 2012 ▶) *T*_min_ = 0.854, *T*_max_ = 1.000
3995 measured reflections
3995 independent reflections
3668 reflections with *I* > 2σ(*I*)

### Refinement  

*R*[*F* ^2^ > 2σ(*F* ^2^)] = 0.053
*wR*(*F* ^2^) = 0.149
*S* = 1.06
3995 reflections
169 parameters
H atoms treated by a mixture of independent and constrained refinement
Δρ_max_ = 0.36 e Å^−3^
Δρ_min_ = −0.27 e Å^−3^

### 

Data collection: *CrysAlis PRO* (Agilent, 2012 ▶); cell refinement: *CrysAlis PRO*; data reduction: *CrysAlis RED*; program(s) used to solve structure: *SUPERFLIP* (Palatinus & Chapuis, 2007 ▶); program(s) used to refine structure: *SHELXL2012* (Sheldrick, 2008 ▶); molecular graphics: *OLEX2* (Dolomanov *et al.*, 2009 ▶); software used to prepare material for publication: *OLEX2*.

## Supplementary Material

Crystal structure: contains datablock(s) I. DOI: 10.1107/S1600536814018972/bt6993sup1.cif

Structure factors: contains datablock(s) I. DOI: 10.1107/S1600536814018972/bt6993Isup2.hkl

Supporting information file. DOI: 10.1107/S1600536814018972/bt6993Isup3.cml

**Fig. 1.**
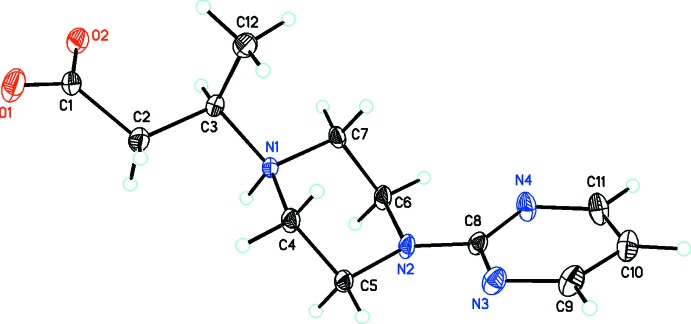
ORTEP drawing of (C12H18N4O2) showing the labeling scheme of the asymmetric unit of the title compound with 30% probability displacement ellipsoids.

**Fig. 2.**
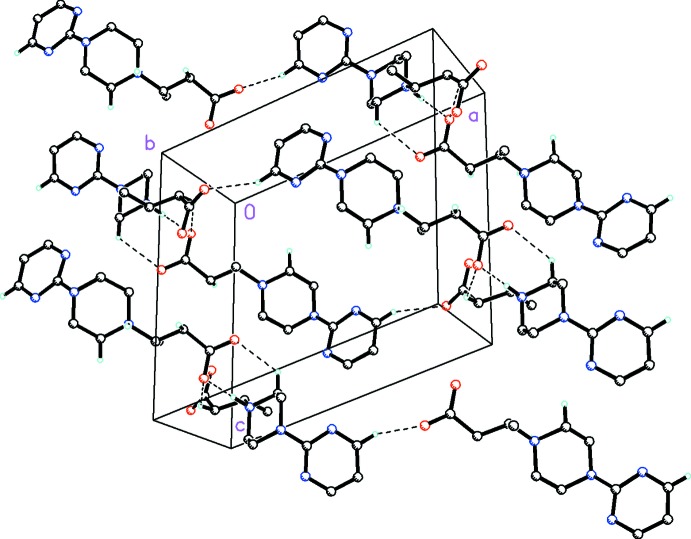
Molecular packing for (I), viewed along the b axis. Dashed lines indicate weak C—H···O intermolecular interactions in addition to N—H···O intermolecular hydrogen bonds which together form an extended three-dimensional supramolecular network structure. H atoms not involved in hydrogen bonding have been removed for clarity.

12 18 4 2 . DOI: 10.1107/S1600536814018972/bt6993fig1.tif
ORTEP drawing of (C_12_H_18_N_4_O_2_) showing the labeling scheme of the asymmetric unit of the title compound with 30% probability displacement ellipsoids.

b . DOI: 10.1107/S1600536814018972/bt6993fig2.tif
Mol­ecular packing for (I), viewed along the *b* axis. Dashed lines indicate weak C—H⋯O inter­molecular inter­actions in addition to N—H⋯O inter­molecular hydrogen bonds which together form an extended three-dimensional supra­molecular network structure. H atoms not involved in hydrogen bonding have been removed for clarity.

CCDC reference: 1020635

Additional supporting information:  crystallographic information; 3D view; checkCIF report

## Figures and Tables

**Table 1:** Hydrogen-bond geometry (Å, °)

*D*—H⋯*A*	*D*—H	H⋯*A*	*D*⋯*A*	*D*—H⋯*A*
N1—H1⋯O2^i^	1.00	1.67	2.653 (2)	168
C2—H2*B*⋯O2^i^	0.99	2.51	3.277 (3)	134
C4—H4*B*⋯O1^ii^	0.99	2.58	3.428 (3)	144
C7—H7*B*⋯O1^i^	0.99	2.53	3.147 (3)	120
C11—H11⋯O1^iii^	0.95	2.47	3.352 (3)	155

Symmetry codes: (i) 
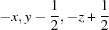
; (ii) 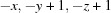; (iii) 
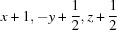
.

## supplementary crystallographic information

### S1. Comment

Pyrimidines in general have been of much interest for biological and medical reasons and thus their chemistry has been investigated extensively (Onal & Yıldırım, 2007) and in many drugs used for the treatment of hypothyroidism and hypertension, in cancer chemotherapy or HIV infections (Burdge, 2000) and with related fused heterocyclic compounds that exhibit biological activities such as anticancer (Amin *et al.*, 2009), antiviral (Ibrahim & El-Metwally, 2010), antibacterial (Kuyper *et al.*, 1996) and antioxidant (Padmaja *et al.*, 2009). Some pyrimidinylpiperazinyl compounds like buspirone and BuSpar (Tollefson *et al.*, 1991) are used to treat anxiety. The incorporation of two moieties increases biological activity of both the molecules. Aza-Michael addition reaction has been extensively studied using a variety of catalysts as well as solvents and various researchers have also reported the utility of aza-Michael addition towards the synthesis of various pharmacological active compounds and proved useful in the synthesis of core intermediates of many natural products (Arend *et al.*, 1998). The role of aza-Michael reaction in the synthesis of pharmacologically important families of β-amino carbonyl compounds and its derivatives is well documented in the literature (Vicario *et al.*, 2005; Xu & Xia, 2005). Our research group has published many papers on incorporated heterocyclic ring structures, viz; 4-(pyrimidin-2-yl)piperazin-1-ium (E)-3-carboxyprop-2-enoate (Yamuna *et al.*, 2014*a*); flupentixol tartarate (Yamuna *et al.*, 2014*b*). Some related zwitterion structures are: 3,3'-(piperazine-1,4-diium-1,4-diyl)dipropionate dihydrate (Jin *et al.*, 2012), enoxacin trihydrate[1-ethyl-6-fluoro-1,4-dihydro-4-oxo-7-(piperazin-4-ium-1-yl)-1,8- naphthyridine-3-carboxylate trihydrate] (Parvez *et al.*, 2004). In view of the importance of derivatives of the incorporated heterocyclic pyrimidylpiperazines, this paper reports the crystal structure of the title zwitterionic compound, (I), 3-(4-Pyrimidin-2-yl-piperazin-1-ium-1-yl)-butanoate, C_12_H_18_N_4_O_2_ prepared from 2-(piperazin-1-yl)pyrimidine and but-2-enoic acid by aza-Michael addition reaction.

The title compound, (I), C_12_H_18_N_4_O_2_ crystallizes in the zwitterionic form with protonation on the N1 nitrogen atom of the piperazine ring (Fig. 1). In the compound, the piperazine ring adopts a slightly disordered chair conformation (puckering parameters Q, θ, and φ = 0.576 (2)Å, 3.0 (2)° and 282 (4)°, respectively. Bond lengths are in normal ranges. In the crystal, N—H···O intermolecular hydrogen bonds are observed forming 1D chains along [0 1 0] (Fig. 2). The packing is consolidated by weak C—H···O interactions which generate a three-dimensional network.

### S2. Experimental

A mixture of 1-(2-pyrimidyl)piperazine, from sigma-aldrich (0.2 g, 1.22 mmol) and crotonic acid (but-2-enoic acid) (0.1048 g, 1.22 mmol ) were dissolved in DMSO, stirred well and warmed at 343 K for 20 minutes. After few days, X-ray quality crystals were obtained on slow evaporation (m.p.: 411-418 K).

### S3. Refinement

H3 was refined isotropically and all of the remaining H atoms were placed in their calculated positions and then refined using the riding model with Atom—H lengths of 0.95Å (CH); 0.99Å (CH_2_); 0.98Å (CH_3_) or 1.00Å (NH). Isotropic displacement parameters for these atoms were set to 1.2 (CH, CH_2_, NH) or 1.5 (CH_3_) times *U*_eq_ of the parent atom. Idealised Me was refined as a rotating group. The title compound was refined as a twin with BASF = 0.41572.

### Crystal data

**Table:**

C_12_H_18_N_4_O_2_	*F*(000) = 536
*M**_r_* = 250.30	*D*_x_ = 1.341 Mg m^−^^3^
Monoclinic, *P*2_1_/*c*	Cu *K*α radiation, λ = 1.54184 Å
*a* = 13.5157 (6) Å	Cell parameters from 5404 reflections
*b* = 7.8454 (3) Å	θ = 3.8–71.4°
*c* = 12.2147 (5) Å	µ = 0.77 mm^−^^1^
β = 106.884 (5)°	*T* = 173 K
*V* = 1239.36 (9) Å^3^	Irregular, colourless
*Z* = 4	0.32 × 0.22 × 0.06 mm

### Data collection

**Table:**

Agilent Eos Gemini diffractometer	3995 independent reflections
Radiation source: Enhance (Cu) X-ray Source	3668 reflections with *I* > 2σ(*I*)
Detector resolution: 16.0416 pixels mm^-1^	θ_max_ = 71.2°, θ_min_ = 3.4°
ω scans	*h* = −16→16
Absorption correction: multi-scan (*CrysAlis PRO* and *CrysAlis RED*; Agilent, 2012)	*k* = −9→9
*T*_min_ = 0.854, *T*_max_ = 1.000	*l* = −13→14
3995 measured reflections	

### Refinement

**Table:**

Refinement on *F*^2^	Primary atom site location: structure-invariant direct methods
Least-squares matrix: full	Hydrogen site location: mixed
*R*[*F*^2^ > 2σ(*F*^2^)] = 0.053	H atoms treated by a mixture of independent and constrained refinement
*wR*(*F*^2^) = 0.149	*w* = 1/[σ^2^(*F*_o_^2^) + (0.0768*P*)^2^ + 0.6386*P*] where *P* = (*F*_o_^2^ + 2*F*_c_^2^)/3
*S* = 1.06	(Δ/σ)_max_ < 0.001
3995 reflections	Δρ_max_ = 0.36 e Å^−^^3^
169 parameters	Δρ_min_ = −0.27 e Å^−^^3^
0 restraints	

### Special details

**Table:**

Geometry. All esds (except the esd in the dihedral angle between two l.s. planes) are estimated using the full covariance matrix. The cell esds are taken into account individually in the estimation of esds in distances, angles and torsion angles; correlations between esds in cell parameters are only used when they are defined by crystal symmetry. An approximate (isotropic) treatment of cell esds is used for estimating esds involving l.s. planes.
Refinement. Refined as a 2-component twin.

### Fractional atomic coordinates and isotropic or equivalent isotropic displacement parameters (Å^2^)

**Table:**

	*x*	*y*	*z*	*U*_iso_*/*U*_eq_	
O1	−0.15848 (13)	0.4680 (3)	0.29107 (16)	0.0449 (5)	
O2	−0.05978 (12)	0.4109 (2)	0.17822 (15)	0.0339 (4)	
N1	0.16438 (12)	0.1694 (2)	0.44116 (14)	0.0217 (4)	
H1	0.1313	0.0618	0.4040	0.026*	
N2	0.32869 (12)	0.0043 (3)	0.60849 (16)	0.0287 (4)	
N3	0.40034 (14)	0.0836 (3)	0.79721 (17)	0.0324 (5)	
N4	0.50457 (13)	0.0549 (3)	0.66957 (17)	0.0336 (5)	
C1	−0.08258 (15)	0.4025 (3)	0.2726 (2)	0.0277 (5)	
C2	−0.00940 (16)	0.2979 (3)	0.3691 (2)	0.0308 (5)	
H2A	−0.0174	0.3357	0.4434	0.037*	
H2B	−0.0296	0.1763	0.3588	0.037*	
C3	0.10322 (15)	0.3150 (3)	0.3722 (2)	0.0260 (5)	
H3	0.098 (2)	0.289 (4)	0.289 (3)	0.041 (8)*	
C4	0.16425 (15)	0.1604 (3)	0.56333 (18)	0.0261 (5)	
H4A	0.0922	0.1532	0.5671	0.031*	
H4B	0.1959	0.2650	0.6040	0.031*	
C5	0.22479 (15)	0.0048 (3)	0.6211 (2)	0.0290 (5)	
H5A	0.2291	0.0053	0.7034	0.035*	
H5B	0.1880	−0.1001	0.5866	0.035*	
C6	0.32772 (15)	0.0092 (3)	0.48941 (19)	0.0276 (5)	
H6A	0.2918	−0.0928	0.4491	0.033*	
H6B	0.3995	0.0084	0.4845	0.033*	
C7	0.27285 (14)	0.1685 (3)	0.43312 (18)	0.0243 (4)	
H7A	0.3102	0.2707	0.4716	0.029*	
H7B	0.2719	0.1716	0.3518	0.029*	
C8	0.41389 (15)	0.0508 (3)	0.69526 (18)	0.0248 (4)	
C9	0.48642 (19)	0.1182 (3)	0.8810 (2)	0.0359 (5)	
H9	0.4802	0.1456	0.9545	0.043*	
C10	0.58388 (18)	0.1162 (3)	0.8665 (2)	0.0372 (6)	
H10	0.6444	0.1349	0.9284	0.045*	
C11	0.58837 (17)	0.0857 (3)	0.7574 (2)	0.0376 (6)	
H11	0.6540	0.0864	0.7436	0.045*	
C12	0.14735 (18)	0.4895 (3)	0.4145 (2)	0.0329 (5)	
H12A	0.1545	0.4997	0.4964	0.049*	
H12B	0.1007	0.5784	0.3725	0.049*	
H12C	0.2153	0.5026	0.4019	0.049*	

### Atomic displacement parameters (Å^2^)

**Table:**

	*U*^11^	*U*^22^	*U*^33^	*U*^12^	*U*^13^	*U*^23^
O1	0.0322 (9)	0.0605 (12)	0.0415 (11)	0.0190 (8)	0.0099 (8)	0.0110 (9)
O2	0.0282 (8)	0.0318 (8)	0.0409 (10)	0.0066 (6)	0.0087 (7)	0.0019 (7)
N1	0.0143 (7)	0.0280 (9)	0.0214 (9)	0.0003 (6)	0.0028 (6)	0.0038 (7)
N2	0.0138 (8)	0.0488 (11)	0.0234 (9)	0.0065 (7)	0.0053 (7)	0.0076 (8)
N3	0.0284 (9)	0.0427 (11)	0.0266 (10)	0.0100 (8)	0.0088 (8)	0.0026 (8)
N4	0.0184 (8)	0.0522 (12)	0.0303 (10)	0.0007 (8)	0.0070 (8)	−0.0065 (9)
C1	0.0200 (9)	0.0303 (11)	0.0295 (11)	−0.0011 (8)	0.0018 (8)	0.0025 (9)
C2	0.0222 (10)	0.0364 (12)	0.0335 (12)	0.0054 (9)	0.0078 (9)	0.0073 (10)
C3	0.0200 (9)	0.0287 (10)	0.0259 (10)	0.0018 (8)	0.0014 (8)	0.0052 (9)
C4	0.0166 (9)	0.0379 (12)	0.0245 (10)	0.0041 (8)	0.0072 (8)	0.0033 (9)
C5	0.0158 (9)	0.0441 (13)	0.0282 (11)	0.0042 (8)	0.0084 (8)	0.0112 (10)
C6	0.0169 (9)	0.0424 (13)	0.0233 (10)	0.0046 (8)	0.0059 (8)	0.0012 (9)
C7	0.0154 (9)	0.0373 (11)	0.0204 (10)	−0.0038 (8)	0.0055 (7)	0.0012 (9)
C8	0.0184 (9)	0.0304 (10)	0.0254 (11)	0.0077 (8)	0.0062 (8)	0.0044 (9)
C9	0.0359 (12)	0.0428 (13)	0.0264 (12)	0.0102 (10)	0.0051 (10)	−0.0020 (10)
C10	0.0297 (11)	0.0408 (13)	0.0336 (13)	0.0035 (10)	−0.0027 (10)	−0.0052 (10)
C11	0.0199 (10)	0.0495 (14)	0.0407 (14)	0.0000 (10)	0.0046 (9)	−0.0103 (11)
C12	0.0322 (11)	0.0287 (11)	0.0337 (13)	−0.0001 (9)	0.0032 (10)	0.0024 (10)

### Geometric parameters (Å, º)

**Table:**

O1—C1	1.226 (3)	C4—H4A	0.9900
O2—C1	1.278 (3)	C4—H4B	0.9900
N1—H1	1.0000	C4—C5	1.524 (3)
N1—C3	1.514 (2)	C5—H5A	0.9900
N1—C4	1.494 (3)	C5—H5B	0.9900
N1—C7	1.498 (2)	C6—H6A	0.9900
N2—C5	1.457 (2)	C6—H6B	0.9900
N2—C6	1.451 (3)	C6—C7	1.513 (3)
N2—C8	1.369 (3)	C7—H7A	0.9900
N3—C8	1.335 (3)	C7—H7B	0.9900
N3—C9	1.335 (3)	C9—H9	0.9500
N4—C8	1.351 (3)	C9—C10	1.379 (3)
N4—C11	1.336 (3)	C10—H10	0.9500
C1—C2	1.538 (3)	C10—C11	1.373 (4)
C2—H2A	0.9900	C11—H11	0.9500
C2—H2B	0.9900	C12—H12A	0.9800
C2—C3	1.518 (3)	C12—H12B	0.9800
C3—H3	1.01 (3)	C12—H12C	0.9800
C3—C12	1.523 (3)		
			
C3—N1—H1	106.5	N2—C5—H5B	109.4
C4—N1—H1	106.5	C4—C5—H5A	109.4
C4—N1—C3	115.63 (17)	C4—C5—H5B	109.4
C4—N1—C7	110.51 (15)	H5A—C5—H5B	108.0
C7—N1—H1	106.5	N2—C6—H6A	109.7
C7—N1—C3	110.70 (15)	N2—C6—H6B	109.7
C6—N2—C5	112.18 (17)	N2—C6—C7	109.75 (18)
C8—N2—C5	122.52 (19)	H6A—C6—H6B	108.2
C8—N2—C6	121.99 (18)	C7—C6—H6A	109.7
C8—N3—C9	115.4 (2)	C7—C6—H6B	109.7
C11—N4—C8	115.6 (2)	N1—C7—C6	109.51 (16)
O1—C1—O2	125.4 (2)	N1—C7—H7A	109.8
O1—C1—C2	118.0 (2)	N1—C7—H7B	109.8
O2—C1—C2	116.65 (19)	C6—C7—H7A	109.8
C1—C2—H2A	109.0	C6—C7—H7B	109.8
C1—C2—H2B	109.0	H7A—C7—H7B	108.2
H2A—C2—H2B	107.8	N3—C8—N2	117.45 (18)
C3—C2—C1	112.91 (18)	N3—C8—N4	126.3 (2)
C3—C2—H2A	109.0	N4—C8—N2	116.26 (19)
C3—C2—H2B	109.0	N3—C9—H9	118.3
N1—C3—C2	109.19 (17)	N3—C9—C10	123.5 (2)
N1—C3—H3	105.9 (16)	C10—C9—H9	118.3
N1—C3—C12	113.11 (17)	C9—C10—H10	122.0
C2—C3—H3	100.5 (16)	C11—C10—C9	116.1 (2)
C2—C3—C12	112.28 (19)	C11—C10—H10	122.0
C12—C3—H3	115.0 (17)	N4—C11—C10	123.0 (2)
N1—C4—H4A	109.6	N4—C11—H11	118.5
N1—C4—H4B	109.6	C10—C11—H11	118.5
N1—C4—C5	110.12 (17)	C3—C12—H12A	109.5
H4A—C4—H4B	108.1	C3—C12—H12B	109.5
C5—C4—H4A	109.6	C3—C12—H12C	109.5
C5—C4—H4B	109.6	H12A—C12—H12B	109.5
N2—C5—C4	110.96 (17)	H12A—C12—H12C	109.5
N2—C5—H5A	109.4	H12B—C12—H12C	109.5
			
O1—C1—C2—C3	−144.6 (2)	C6—N2—C5—C4	−57.3 (3)
O2—C1—C2—C3	36.8 (3)	C6—N2—C8—N3	163.5 (2)
N1—C4—C5—N2	54.5 (2)	C6—N2—C8—N4	−18.1 (3)
N2—C6—C7—N1	−59.2 (2)	C7—N1—C3—C2	172.41 (18)
N3—C9—C10—C11	3.7 (4)	C7—N1—C3—C12	−61.8 (2)
C1—C2—C3—N1	−161.91 (18)	C7—N1—C4—C5	−55.5 (2)
C1—C2—C3—C12	71.8 (3)	C8—N2—C5—C4	102.5 (2)
C3—N1—C4—C5	177.77 (16)	C8—N2—C6—C7	−100.4 (2)
C3—N1—C7—C6	−172.54 (17)	C8—N3—C9—C10	−1.8 (4)
C4—N1—C3—C2	−61.0 (2)	C8—N4—C11—C10	−1.8 (4)
C4—N1—C3—C12	64.8 (2)	C9—N3—C8—N2	176.1 (2)
C4—N1—C7—C6	58.0 (2)	C9—N3—C8—N4	−2.2 (3)
C5—N2—C6—C7	59.5 (2)	C9—C10—C11—N4	−1.7 (4)
C5—N2—C8—N3	5.7 (3)	C11—N4—C8—N2	−174.3 (2)
C5—N2—C8—N4	−175.9 (2)	C11—N4—C8—N3	4.0 (4)

### Hydrogen-bond geometry (Å, º)

**Table:**

*D*—H···*A*	*D*—H	H···*A*	*D*···*A*	*D*—H···*A*
N1—H1···O2^i^	1.00	1.67	2.653 (2)	168
C2—H2*B*···O2^i^	0.99	2.51	3.277 (3)	134
C4—H4*B*···O1^ii^	0.99	2.58	3.428 (3)	144
C7—H7*B*···O1^i^	0.99	2.53	3.147 (3)	120
C11—H11···O1^iii^	0.95	2.47	3.352 (3)	155

Symmetry codes: (i) −*x*, *y*−1/2, −*z*+1/2; (ii) −*x*, −*y*+1, −*z*+1; (iii) *x*+1, −*y*+1/2, *z*+1/2.
