# Supplementary figures and images for: Crystal structure of 3-[4-(pyrimidin-2-yl)piperazin-1-ium-1-yl]butano­ate
Source: Acta Crystallogr Sect E Struct Rep Online. 2014 Aug 30;70(Pt 9):o1063–4. doi: 10.1107/S1600536814018972 (PMC4186175; doi:10.1107/S1600536814018972)

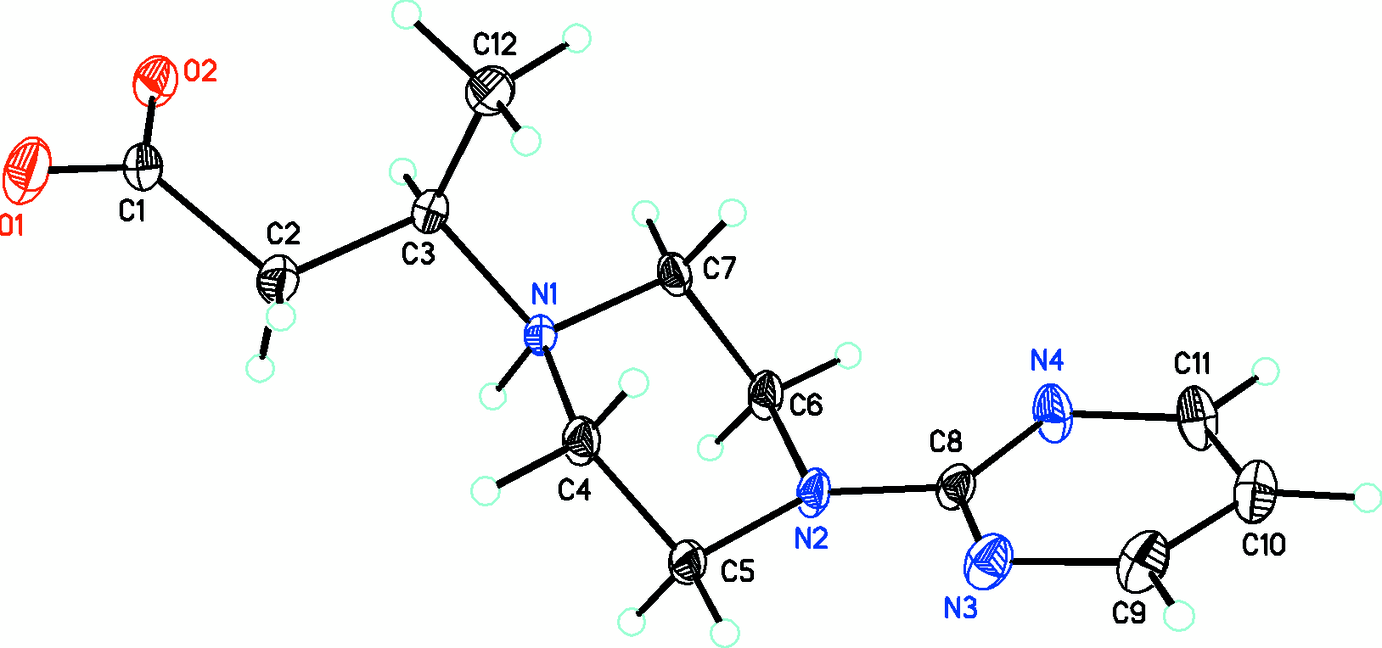

Supplement: Supplementary file 4 [file e-70-o1063-fig1.tif]

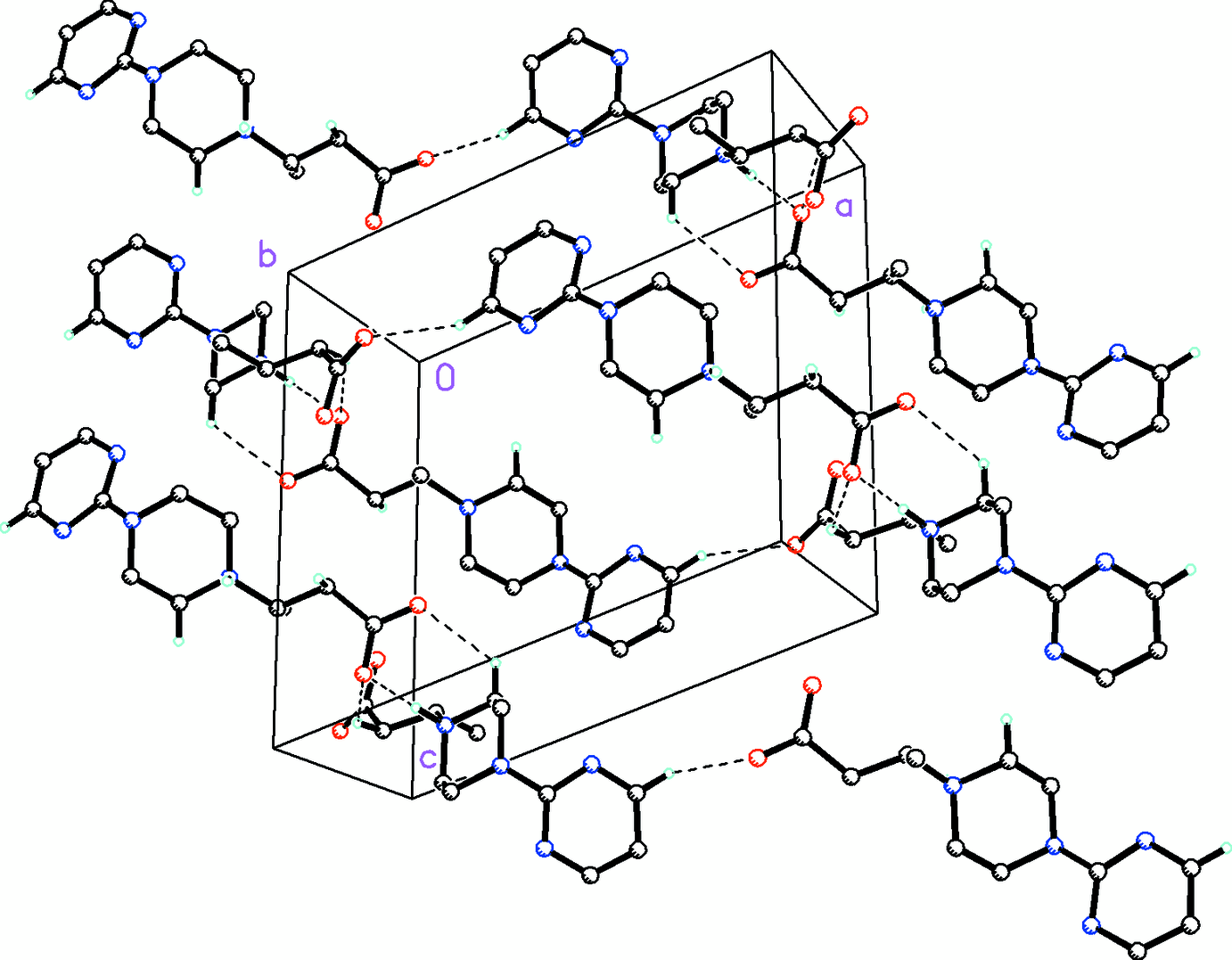

Supplement: Supplementary file 5 [file e-70-o1063-fig2.tif]
